# Adenolipoleiomyoma Polyp of the Uterus: A Case Report and Review of the Literature

**DOI:** 10.1155/2018/5704382

**Published:** 2018-09-06

**Authors:** Xavier Catteau, Vincent Anaf, Jean-Christophe Noël

**Affiliations:** ^1^CUREPATH, Rue de Borfilet 12A, 6040 Jumet, Belgium; ^2^Pathology Department, Erasme University Hospital, Université Libre de Bruxelles, CP 610, Route de Lennik 808, 1070 Brussels, Belgium; ^3^Gynaecology Department, Erasme University Hospital, Université Libre de Bruxelles, CP 610, Route de Lennik 808, 1070 Brussels, Belgium

## Abstract

Adenolipoleiomyoma is a very rare lesion of the uterus. Only four cases were reported. We describe one case of adenolipoleiomyoma presenting as a polyp in a postmenopausal woman with menorrhagia. Adenolipoleiomyoma is a very rare lesion and the histogenesis remains unclear. We discuss the origin and the differential diagnosis of this lesion.

## 1. Introduction

An adenolipoleiomyoma is a very rare lesion of the uterus. This is a type of adenomyomatous polyp that contains müllerian-type glands, smooth muscle, and adipose tissue. Only four reports of adenolipoleiomyoma have been described [1–4]. One of them had an aggressive behaviour with recurrence [1].

## 2. Case Presentation

**Figure 1:**
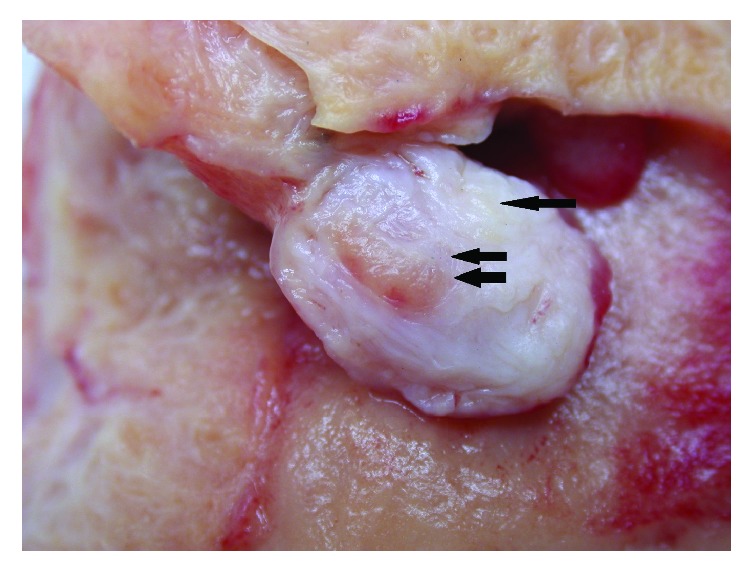
Macroscopically, this polyp appeared white, fibrous, fasciculate (glandular component, double arrow), and focally yellow (one arrow).

**Figure 2:**
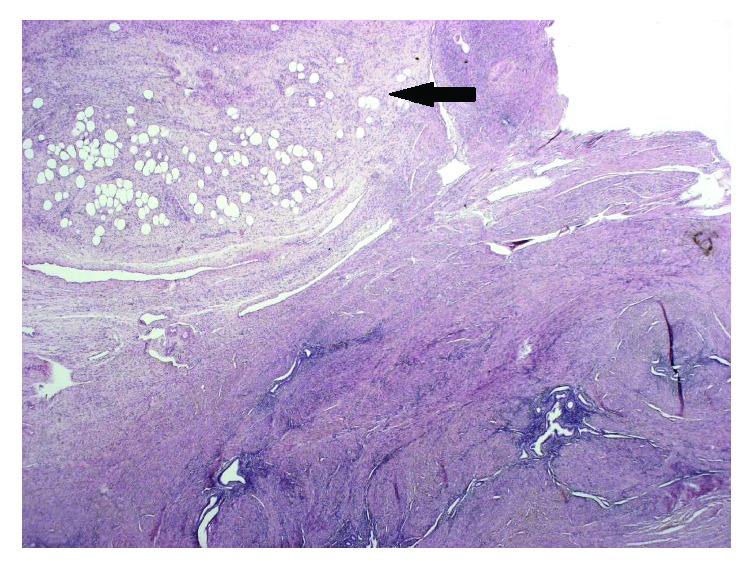
Lesion at low power-view with smooth muscle, glands, and adipocytes (one arrow) components. Lesion with adipocytic (center of the lesion, arrow) and glandular components (X20).

This is a fifty-five-year-old woman with past appendectomy. She was perimenopausal and had no treatment. Her brother had a rectum neoplasia. She had four children. She suffered from anemia because she had menorrhagia since few months. Ultrasonography examination revealed a polymyomatous uterus and a vascularized polyp of 23x17 mm in the uterine cavity. Mammography and breast ultrasonography were normal. CA125 was high (63,3 U/ml). Total hysterectomy with bilateral ovariectomy was performed because its symptoms lasted for too long. Histological examination showed a subatrophic endometrium with bands of irregular proliferation, slight-to-moderate adenomyosis, and benign subserosal leiomyoma of 34 mm in size. There was also a pedunculated polyp of 25 mm. Macroscopically, this polyp appeared white, fibrous, fasciculated, and focally yellow (Figure 1). Microscopically, we observed endometrial glands with a stroma composed of smooth muscle and adipocytes (Figure 2). The zone appearing macroscopically fatty contained mature adipose tissue. No atypia was observed. The adipocytes were positive for estrogen and progesterone receptor. A diagnosis of adenolipoleiomyoma was made. No specific particularities were revealed in the follow-up at two years.

## 3. Discussion

Adenolipoleiomyoma is a rare polypoid uterine lesion composed of müllerian-type glands (glands of müllerian origin like endometrium), smooth muscle, and adipose tissue. The histogenesis of this lesion remains controversial.

Mc Cluggage et al. and Payne et al. believe that uterine adenolipoleiomyoma is a benign hamartomatous lesion. They think that lesion represents a hamartomatous lesion with adipocytic metaplasia, a sort of direct metaplasia from a preexisting tissue present normally in the uterus [2, 3].

Yavuz suggests that it is a variant of benign müllerian mixed tumour [1]. Ridvan et al. think that their case presents features of hamartoma: adipose tissue and thick-walled vascular structures intensely admixed and the absence of internal elastic lamina in the arteriolar structures [5]. However, they described cases of müllerian adenosarcoma and rhabdomyosarcoma of the uterine cervix with heterologous mesenchymal elements, such as cartilage and fatty tissue. They think that this type of lesion is a müllerian-type lesion, because müllerian stromal cells have the capacity to be transformed into other mesenchymal-type cells [5]. The case described by Shaco-Levy et al. with an aggressive behaviour could be an atypical müllerian lesion rather than a hamartoma. In our case, we think that is a hamartoma with direct adipocytic metaplasia. The fact that no atypia was observed and the presence of benign endometrial glands and smooth muscle are in support of a hamartomatous origin. Nevertheless, our case is the only one associated with adenomyosis and a differential diagnosis must be made between adenolipoleiomyoma and lipoleiomyoma involved by adenomyosis. In our case, a well-demarcated lesion with central fatty zone is an argument for adenolipoleiomyoma (Figure 1). The slight elevation of Ca 125 may be due to adenomyosis but this data is not clearly found in the literature. No association between adenolipoleiomyoma and Ca 125 was reported.

In the literature, the mean age is 45.8 years and the mean size of tumour is 5.2 cm. One case has been described in cervix [4]. Hysterectomy is the most frequent treatment but depends on the age of the patient.  Table 1 summarizes the cases described in literature.

Differential diagnoses are

1. atypical polypoid adenomyoma that contains an architecturally complex and cytological atypical glandular component, whereas adenomyomatous polyp has simple glands and no significant atypia [6];
2. lipoleiomyoma involved by adenomyosis: in this type of lesion the structures are not clearly mixed;
3. adenomyoma with adipocytic metaplasia that differs from adenolipoleiomyoma by the aspect of adipocytes (more irregular);
4. benign variant of mixed müllerian tumour that contains very rarely three components;
5. adenosarcoma that exhibits a more complex pattern of spindle cells with mitoses;
6. common uterine leiomyoma that contains no glandular component [1, 7].

## 4. Conclusion

Adenolipoleiomyoma is very rare and the histogenesis of this lesion remains unclear. Although this tumour seems to be most frequently benign, prolonged follow-up will be necessary to confirm this benignity [2].

## Figures and Tables

**Table 1:** Clinical and pathological features of the current tumor and uterine adenolipoleiomyoma reported in the literature.

Authors	Age (yr)	Presentation	Tumor location	Tumor largest diameter (cm)	Histology	Therapy	Outcome, Follow-up
Payne et al.	19	Irregular vaginal bleeding	Polypoid endometrial mass	2.5	Smooth muscle, endometrial stroma, adipose tissue, endometrioid-type epithelium	Polypectomy	NED*∗*, 1 yr

McCluggage et al.	53	Incidental finding in a patient with retrovaginal prolapse	Intramural uterine mass	2.5	Smooth muscle, endometrial stroma, adipose tissue, endometrioid, endocervical and tubal-type epithelium	Hysterectomy	None

Yavus et al.	52	Lower abdominal pain and postmenopausal bleeding	Polypoid endometrial mass	7.0	Smooth muscle, endometrial stroma, adipose tissue, endometrioid-type epithelium	Hysterectomy and bilateral salpingo-oophorectomy	None

Shaco-Levy et al.	40	Pelvic mass.	Intramural and subserosal masses	1st resection: 16 2^nd^ resection: 43	First resection: smooth muscle, adipose tissue, endocervical-type epithelium Second resection: smooth muscle, adipose tissue, endometrial-type stroma, endocervical-type epithelium, focally atypically proliferating, and endometrioid-type epithelium	1^st^ resection: conservative excision masses 2^nd^ resection: total hysterectomy, bilateral salpingo-oophorectomy and resection of tumor masses.	Local recurrence after 16 months Second resection: NED after 4 months

Current case.	55	Irregular vaginal bleeding	Intraluminal uterine polyp	2.5	Smooth muscle, adipose tissue, endometrioid-type epithelium	Total hysterectomy, bilateral salpingo-oophorectomy	NED, 6 months

*∗*NED indicates no evidence of disease.
